# Combining photocatalytic hydrogen generation and capsule storage in graphene based sandwich structures

**DOI:** 10.1038/ncomms16049

**Published:** 2017-07-06

**Authors:** Li Yang, Xiyu Li, Guozhen Zhang, Peng Cui, Xijun Wang, Xiang Jiang, Jin Zhao, Yi Luo, Jun Jiang

**Affiliations:** 1 Hefei National Laboratory for Physical Sciences at the Microscale, iChEM (Collaborative Innovation Center of Chemistry for Energy Materials), CAS Key Laboratory of Mechanical Behavior and Design of Materials, School of Chemistry and Materials Science, University of Science and Technology of China, Hefei, Anhui 230026, China; 2 ICQD/Hefei National Laboratory for Physical Sciences at the Microscale, and Key Laboratory of Strongly-Coupled Quantum Matter Physics, Chinese Academy of Sciences, and Department of Physics, University of Science and Technology of China, Hefei, Anhui 230026, China; 3 Synergetic Innovation Center of Quantum Information & Quantum Physics, University of Science and Technology of China, Hefei, Anhui 230026, China

## Abstract

The challenge of safe hydrogen storage has limited the practical application of solar-driven photocatalytic water splitting. It is hard to isolate hydrogen from oxygen products during water splitting to avoid unwanted reverse reaction or explosion. Here we propose a multi-layer structure where a carbon nitride is sandwiched between two graphene sheets modified by different functional groups. First-principles simulations demonstrate that such a system can harvest light and deliver photo-generated holes to the outer graphene-based sheets for water splitting and proton generation. Driven by electrostatic attraction, protons penetrate through graphene to react with electrons on the inner carbon nitride to generate hydrogen molecule. The produced hydrogen is completely isolated and stored with a high-density level within the sandwich, as no molecules could migrate through graphene. The ability of integrating photocatalytic hydrogen generation and safe capsule storage has made the sandwich system an exciting candidate for realistic solar and hydrogen energy utilization.

## Abstract (web summary)

Photocatalytic water splitting is an attractive route to hydrogen production, but safely isolating hydrogen and oxygen products remains a challenge. Here, the authors propose a carbon nitride/grapheme (GR)-based sandwich structure, providing safe and effective photocatalytic hydrogen generation and capsule storage.

Aiming to develop clean and sustainable energy resource, photocatalytic water splitting has gained great interests in both academia and industry communities^1^,^2^,^3^. By harvesting solar light, a photocatalyst generates energetic charges to split water into hydrogen (H_2_) and oxygen (O_2_) molecules^1^, through which solar power is converted to hydrogen power, a clean and high calorific energy resource. Over years, many promising photocatalysts have been made, such as metal oxides or sulfides^4^,^5^,^6^,^7^, metal organic frameworks^8^, pure metals^9^ and metal-free semiconductors^10^ and so on. Recently, GR-based non-metallic photocatalysts exhibited advantages in both high performance and low cost^11^,^12^,^13^,^14^. For instance, g-C_3_N_4_, a graphitic carbon nitride, offers good photocatalytic performance together with thermodynamic stability and engineerability^11^. A recent work using nano-composite photocatalyst of g-C_3_N_4_ and carbon nanodot achieved solar energy conversion efficiency of 2.0% for water splitting^12^.

Unfortunately, the future of widely utilizing hydrogen energy generated from sustainable solar and water resources is hampered by the difficulty of hydrogen collection and storage. Hydrogen generation relies on the delivery of photo-generated electron and hole charges to the photocatalyst reductive and oxidative sites, respectively. To enable energetic carriers driving reactions, the distance between reductive and oxidative sites is limited to the charge migration length (electron mean free path in 10–50 nm). Moreover, protons produced at oxidative sites need to migrate to reductive sites for H_2_ generation, which also requires short reductive–oxidative distance. However, the short reductive–oxidative distance not only promotes unwanted reverse reactions (recombining proton and –OH/O_2_ to water) that seriously reduce the efficiency, but also leads to extreme difficulties to collect or store H_2_ without oxygen contamination. In practice, hydrogen storage is known to be a technical challenge. The mixture of H_2_ with O_2_ easily causes dangerous explosion, while metal or carbon fibre containers widely used for H_2_ storage are expensive. Thus, the practical utilization of photocatalytic water splitting could not be realized until a cost-effective solution is developed to completely isolate hydrogen from oxygen during reactions and safely store H_2_ afterwards.

Meanwhile, the technique advancements^15^,^16^,^17^ in fabricating large-area GR and GR oxide (GO: GR modified by hydroxyl and epoxy groups) make the capsule of an appreciable amount of H_2_ feasible. GR-based materials have exhibited excellent hydrogen-adsorption ability with relatively low cost compared with widely used metal materials. For instance, by combining palladium and activated carbon with GR, Lee and colleagues^18^ achieved high performance in hydrogen storage capacity reaching 0.82 wt% (by weight) at ∼8 MPa (improving nearly 49% compared with pure palladium). Urban and colleagues^19^ constructed an environmentally stable GO and Mg nanocrystal composite with atomic thickness, which accomplishes hydrogen storage of 6.5 wt%.

Recently, hetero-structures based on two-dimensional (2D) nanosheets have also attracted great attention owing to excellent properties and fabrication feasibility^20^,^21^. Xie and colleagues^22^ constructed a model of GR confined ultrathin tin quantum sheets, achieving high electrochemical activity and excellent stability for carbon dioxide radical anion. As a metal-free system, GR–C_3_N_4_ composite layers can effectively harvest visible solar light^14^. The GR-based materials hold high mobility in delivering charges towards efficient electrochemical and photochemical reactions^23^,^24^. Efficient electron–hole separation could be achieved through ultrafast charge transferring between GR–C_3_N_4_ layers despite of their weak van der Waals interaction, which has been experimentally observed in vertically stacked transition-metal dichalcogenide heterostructure bilayers with weak van der Waals interaction^25^,^26^,^27^. Moreover, functional groups on GR, including hydroxyl and epoxy, doped heteroatoms, and defects, provide good active sites for water decomposition^20^,^28^,^29^,^30^,^31^,^32^. Our theoretical and experimental works demonstrate that parts of photo-generated electrons in carbon nitrides are collected at nitrogen positions^33^,^34^, providing ideal reductive sites for hydrogen generation^35^. Moreover, Geim, Wu and colleagues^36^ have demonstrated that GR film is not permeable for any particle larger than proton (or hydrogen atom), and the penetration of proton would be particularly efficient if driven by electrostatic attractions between protons and negative-charged ions.

Therefore, we can anticipate that a proper use of all these remarkable properties of GR-based materials can lead to a strategy to utilize the safe storage of hydrogen during the water splitting. For this purpose, here we design a multi-layer structure of C_*x*_N_*y*_ and GR network, where a graphitic carbon nitride (g-C_*x*_N_*y*_) layer is sandwiched between two GR layers modified by functional groups (GR_F_). It takes advantage of the high efficiency of GR_F_–C_*x*_N_*y*_ for photocatalytic water splitting, and the strict mass transport selectivity of GR to achieve safe capsule H_2_ storage. Based on three known graphitic carbon nitride structures of CN, C_2_N, C_3_N_4_ (Fig. 1a), we build the model sandwich system GR_F_–C_*x*_N_*y*_–GR_F_ (Fig. 1b, where g-CN and GO are used as prototype). The water splitting and hydrogen capsuling scheme is illustrated in Fig. 1b: (1) by absorbing visible or ultraviolet light, GO–C_*x*_N_*y*_ generates excitons, which soon separate to energetic electrons and holes. Specifically, electrons on the inner g-C_*x*_N_*y*_ mainly localized on N sites, while holes transfer to two outer GO layers, according to different material properties. (2) The holes on GO attack water molecules adsorbed on GO functional sites, triggering water splitting to produce protons (H^+^). (3) Driven by electrostatic attractions, protons penetrate through GO to meet electrons on N sites of g-C_*x*_N_*y*_, and consequently produce H_2_ molecules. (4) Since H_2_ cannot transport through GR or GR_F_ (GO), it would be retained in between two GO layers, realizing the purpose of capsule storage. In view of the above processes, a series of related calculations are carried out, including charge separations, chemical reactions, and H_2_ storages. Our calculation results confirm the rationality and feasibility of integrating hydrogen production and capsule storage into one system. We study the electronic structures and couplings of GR network with g-C_*x*_N_*y*_ in the hybrid structures (Supplementary Fig. 1), to examine their stability, photo-absorption and charge distributions. Reactions of water splitting (oxidative) and hydrogen generation (reductive) are then investigated based on isolated monolayers of GR_F_ sheet and g-C_*x*_N_*y*_, respectively. Hydrogen storage ability is studied with the GR–C_*x*_N_*y*_–GR sandwich model.

## Results

### Photo-generated electrons and holes separation

For all optimized sandwich structures, g-C_*x*_N_*y*_ and GR/GO layers exhibit similar interfacial distances of 2.94–3.26 Å and adhesion energies from 1.02 to 3.73 eV (Supplementary Table 1), suggesting good structural stability. The interfacial distance and adhesion energy between g-C_3_N_4_ and GR are about 3.09 Å and 1.04 eV, agreeing well with the previous report^14^. The simulated electronic structures confirmed the semiconductor feature of g-C_*x*_N_*y*_, which enables photon energy harvesting. The computed dielectric function suggested that bare CN, C_2_N, C_3_N_4_ mainly absorb ultraviolet light (Supplementary Fig. 2), in consistent with their band energy gaps (Supplementary Fig. 3). Their gaps were narrowed by coupling with GR/GO layers (Supplementary Fig. 3)^14^, enabling the hybrid systems to harvest both visible and ultraviolet photons (Supplementary Fig. 2), and thereby convert the majority of solar power into energized electrons and holes.

**Figure 2:**
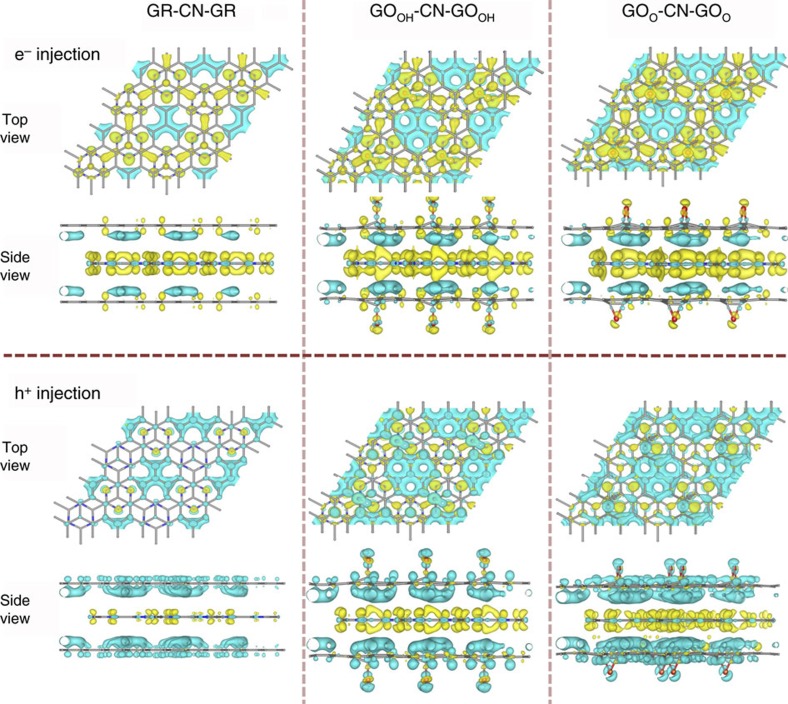
Photo-generated carrier distributions in the sandwich structures. Charge distribution computed as Bader charge differences between GR/GO–CN–GR/GO sandwich with one extra carrier (photo-generated electron (e^−^) or hole (h^+^)) and the neutral monolayers of GR/GO and g-CN, from top and side view. Yellow and blue bubbles represent electron and hole charges with isosurface value of 0.0005, e Å^−3^.

The next key step is to separately deliver photo-generated electrons and holes to reductive and oxidative reaction sites. It is well documented that a difference in work function between different materials could induce electron flowing from the material with lower work function to the one with higher work function^37^. Here we found that the GR-based material has lower work functions than the C_*x*_N_*y*_, ranging from 1.7 to 2.91 eV (Supplementary Fig. 4). Therefore, before the photo-excitation, the sandwiched g-CN, C_2_N and C_3_N_4_ unit cells could donate 0.34–0.72, 0.18–0.20 and 0.16–0.25 positive charges (h^+^) to the outer GR/GO layers, respectively (Table 1, Supplementary Fig. 5), exhibiting well-separated electron and hole carrier distributions. We also carried out time dependent *ab initio* non-adiabatic molecular dynamics (AI-NAMD) simulations to describe the ultrafast hole evolution process. In Supplementary Fig. 6, it is shown that the hole with lower energy (near the valence band) could transfer from CN to GR, and ∼14% hole carriers would reach the GR sheet within 3 ps. While for the hole of higher energy, more than half of the carriers would quickly transfer to the GR sheet within 0.4 ps. Ultimately, above 80% hole carriers in CN layer would transfer to the GR layer within a few ps. Such an ultrafast charge transfer can compete with the electron-hole recombination and ensure the subsequent reactions (that is, water splitting on GO, and H_2_ generation on C_*x*_N_*y*_). By adding 1.0 extra electron or hole carrier to the sandwich system, we have modelled the distributions of photo-generated charges. As displayed in Fig. 2 and Supplementary Fig. 7 and 8), the outer GR or GO layers always collect hole carriers, whereas inner g-C_*x*_N_*y*_ accumulates electrons. In Table 1, one photo-generated electron carrier induces about 0.68–0.87, 0.33–0.45 and 0.32–0.33 e^−^ in the inner CN, C_2_N and C_3_N_4_ units, respectively. Although the GR/GO cells can collect 1.17–1.59 (with CN), 1.01–1.10 (with C_2_N) and 1.01–1.23 (with C_3_N_4_) h^+^ if one extra hole was injected, suggesting photo-generated holes on the outer GO layers.

### Water splitting driven by photo-induced energetic charges

**Figure 3:**
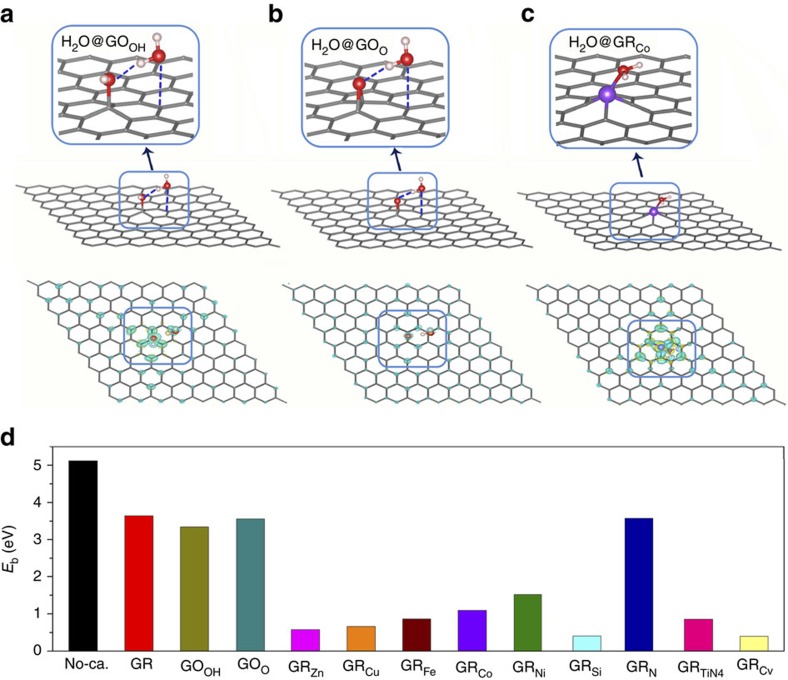
Water splitting and proton generation at the outer GR_F_ surface. (**a**–**c**) Optimized H_2_O adsorption geometries and charge distributions (with one photo-generated h^+^) on GO_OH_, GO_O_ and GR_Co_ sheets. (**d**) The computed energy barrier (*E*_b_) of water splitting reaction catalysed by GR or GR_F_. Here data of pure GR are retrieved from ref. 30, and No-ca. stands for bare water reaction without catalyst. GO_OH_ and GO_O_ represent the hydroxyl and epoxy GO; GR_Zn_, GR_Cu_, GR_Ee_, GR_Co_, GR_Ni_ represent the metal-doped GR with Zn, Cu, Fe, Co, Ni atom, respectively; GR_Si_, GR_N_ stand for Si, N atom-doped GR; GR_TiN4_ represents TiN_4_-doped GR and GR_Cv_ means for GR with a carbon defect. Yellow and blue bubbles represent electron and hole charges with isosurface value of 0.0005, e Å^−3^. The observation windows in **a**–**c** highlight the local adsorption sites for water molecule.

The water splitting starts with H_2_O adsorption to the outer GR_F_ surfaces. Functional groups on GR induce polarized charges around active sites, which is helpful for H_2_O adsorption (Fig. 3a–c). Relatively high adsorption energies were found for water on various GR_F_ materials (*E*_ads_ in Supplementary Table 2), with 0.32–0.37 eV for GO (Supplementary Fig. 9), 0.60–1.02 eV for GR_M_ (metal: Zn, Cu, Fe, Co, Ni in Supplementary Fig. 10), 0.47 and 0.07 eV for GR_Si_ and GR_N_ (Supplementary Fig. 11), 1.05 eV for GR_TiN4_ (TiN_4_ on GR in Supplementary Fig. 12), and 0.20 eV for GR_Cv_ (GR with a carbon defect C_v_ in Supplementary Fig. 13), respectively. Noticeably, it is shown in Fig. 3a–c that functional groups could collect much hole carriers after adsorbing water molecule, making it ready for oxidative reaction. Moreover, these groups effectively reduce the energy barrier (*E*_b_) for water splitting. Based on climbing image nudged elastic band calculations for transition states (Supplementary Figs 9–13), the *E*_b_ value of 5.13 eV for bare H_2_O is decreased to 3.64 eV with the GR catalyst, 3.34–3.56 for the GO_OH_ and GO_O_ catalysts, 0.58–1.09 eV for the GR_M_ (metal: Zn, Cu, Fe, Co), 0.41 eV for the GR_Si_, 0.86 eV for the GR_TiN4_ and 0.40 eV for the GR_Cv_ (Supplementary Table 2). During photocatalysis, such energy barriers could be easily overcome by GR_F_ receiving photo-induced energetic hole carriers. Similar to GO/GR, GR_F_ with other functional groups tend to extract positive charges from the middle C_*x*_N_*y*_ layer (Supplementary Fig. 14 and Supplementary Table 3), validating the feasibility of photo-generated charge separation. The hole injection effectively reduced the Gibbs free energy for water splitting from 1.55 and 2.34 eV at the neutral state to 1.08 and 1.67 eV for the positively charged GO_OH_ and GO_O_, respectively, favouring water splitting in terms of thermodynamics (Supplementary Table 4).

### Protons penetration and hydrogen generation

**Figure 4:**
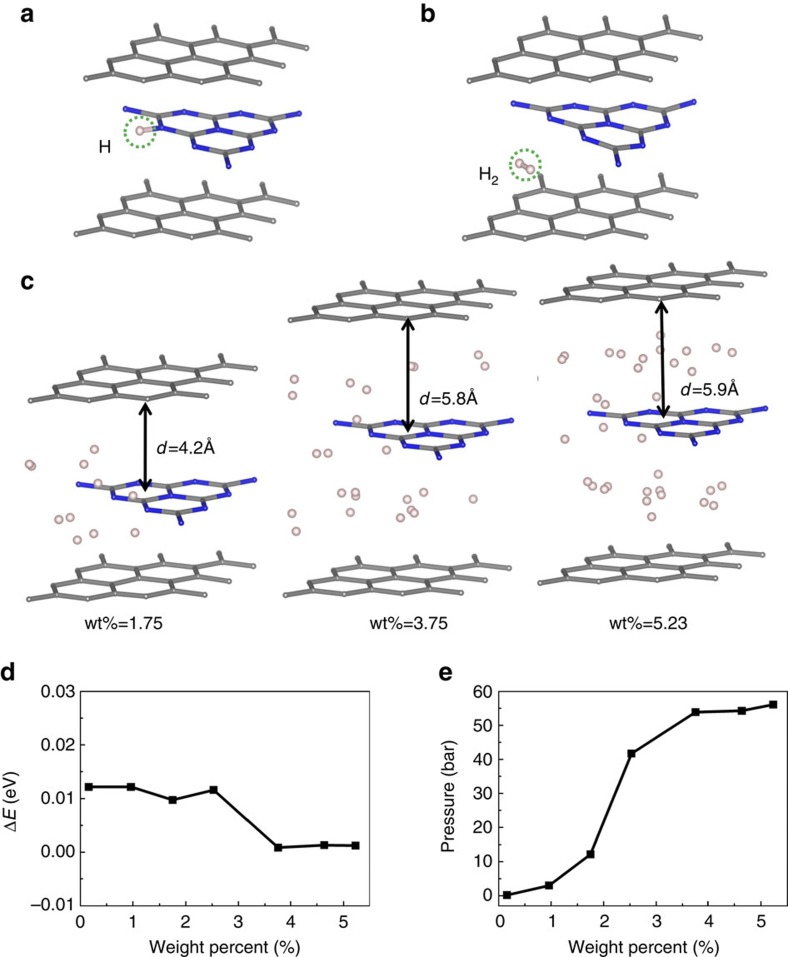
Hydrogen evolution and capsule storage. (**a**–**c**) Optimized configurations of the GR–C_3_N_4_–GR sandwich adsorbed with one H atom, one H_2_ molecule and many H_2_ molecules with different interfacial spaces. *d* in **c** stands for interfacial distance. (**d**) The energy costs (Δ*E*) to achieve different H_2_ store rate. (**e**) The variation of the pressure imposed to the outer graphene sheet for GR–C_3_N_4_–GR sandwich structure to achieve different H_2_ store rate. 1 bar=10^5^ Pa (∼1 standard atmospheric pressure).

Driven by the electrostatic attraction force, protons produced at the oxidative sites would penetrate through the outer GR-based layers to meet the inner g-C_*x*_N_*y*_. The electrostatic interaction energy between the proton and the C_*x*_N_*y*_ with photo-generated electrons is 1.48–4.04 eV, which could readily overcome the proton transport barrier in GR of ∼1.23 eV (ref. 36) (Supplementary Table 5). This process is also verified by our *ab initio* MD simulations for proton transfer through the GR sheet in the GR-C_3_N_4_ structure (Supplementary Movie 1, Supplementary Fig. 15), in which parts of photo-generated electrons could be collected by nitrogens. As shown in Fig. 4a, the proton would be chemically bonded with nitrogen. Bader charge analysis found that such N–H bond extracts ∼10% of one photo-generated e^−^ in the whole GR–C_3_N_4_–GR unit. The negative charge can attract another approaching proton, which consequently bonds to the previous H to generate a H_2_ molecule. The g-C_*x*_N_*y*_ desorbs H_2_ (Fig. 4b) exothermically, with 0.17 eV for neutral structure while 0.25 eV for negative charged one. Geim and colleagues^36^ have demonstrated that GR network allows only proton to pass through. Therefore, O_2_ and hydroxyl radicals are kept outside from the inside H_2_ and protons (attracted by negative charges). These thus suppress the unwanted reverse reaction in water splitting, and realize the complete hydrogen evolution process.

### Capsule hydrogen storage

With sufficient energetic electrons and holes supplies from photo-excited g-C_*x*_N_*y*_, appreciable amounts of H_2_ molecules would be produced. As H_2_ cannot pass through GR-based material, it would be capsuled inside the sandwich. The commercial H_2_ storage standard postulated by US Department of Energy is 65 kg m^−3^ (hydrogen storage system per se) and 6.5 wt% (by weight)^38^. Although pressurization techniques, container ameliorations and extra absorbents could help to reach this goal, large-scale storage and transportation were limited due to high cost, security risk and technique difficulty. While our sandwich structure can safely capsule H_2_. We have tested the accommodations of different amounts of H_2_ in the GR–C_3_N_4_–GR sandwich (Fig. 4c, Supplementary Fig. 16), by allowing whole structure relaxation. The interfacial distance increases from 3.0 to 5.5 Å with the store rate from zero to 2.5 wt%, and saturates at 5.5–5.9 Å with store rate from 2.5 to 5.2 wt% (Supplementary Fig. 17). The storage causes almost no structure deformations, and requires nearly negligible energy with 0.001–0.012 eV for 0.2–5.23 wt% store rate (Fig. 4d). In the distance range of 3.6–5.9 Å, effective interlayer couplings still exist and thereby enable ultraviolet–vis light-absorption and hole charge transfer (Supplementary Fig. 18 and Supplementary Table 6). These agree with previous experimental reports on effective charge transfer between 2D material with interlayer distance of 6–7 Å (refs 25, 27). Moreover, the use of pressurization can easily maintain high H_2_ store rate by controlling interfacial distance. We have found that the interfacial distance can be maintained at ∼3.1 Å by imposing a certain amount of pressure to the outer GR sheet (Fig. 4e). For example, the storage rate of 5.2 wt% can be achieved with ∼3.1 Å interfacial distance by adding ∼56 bar (1 bar=10^5^ Pa≈1 atm) external pressure, which is comparable with conditions for other storage systems reported in literature (Supplementary Fig. 19). The rate of 5.2 wt% is close to the standard (6.5 wt%) proposed by US DOE. Although our storage rate is yet to reach the best reported values^39^, our design holds unique advantages, such as low cost, and the combination of both hydrogen production and generation.

## Discussion

In summary, we designed the sandwich structure of one g-C_*x*_N_*y*_ in between two GR_F_, to achieve efficient solar harvest, charge separation and reverse reaction inhibition for photocatalytic water splitting, and more importantly to integrate simultaneous hydrogen generation and capsule storage in a single system. It harvests visible and ultraviolet light to generate energetic holes and electrons distributing separately on oxidative and reductive sites of GR_F_ and g-C_*x*_N_*y*_. After water splitting on GR_F_ surface, protons penetrate it through to produce H_2_ inside the sandwich. By allowing only protons to pass through the outer GR layers, it not only suppresses unwanted reverse reaction, but also capsules H_2_ products to achieve safe storage. Exploring sp^2^-carbon sheets (GR, fullerene, carbon-nanotube) to capsule many other promising photocatalysts in the same framework would concrete this sandwich concept and provide better candidates for applications. This would stimulate an alternative way of thinking for water splitting towards realistic solar and hydrogen energy utilizations.

## Methods

### Structural optimization and analysis

All calculations were performed by the Vienna Ab initio Simulation Package at the density functional theory level^40^. Generalized gradient approximation^41^, Perdew, Burke, and Ernzerh functional together with projector augmented-wave pseudopotential^42^ were employed. State-of-the-art hybrid functional (HSE06) was used for band structure, work function and absorption spectrum calculations^43^. Van der Waals correction was included to account for the nonbonding interaction between GR-based layers and g-C_*x*_N_*y*_. The kinetic energy cutoff was set to be 400 eV in the plane-wave expansion. The periodic boundary condition was set with a 15 Å vacuum region above the plane of GR sheet. For the supercell structure, the Morkhost pack mesh of *K* points was 3 × 3 × 1, while that for single cell was 9 × 9 × 1.

### Transition state calculations

Climbing image nudged elastic band^44^ was used for the search of transition states, and these states were validated by the SSW package HOWTOs program^45^.

### Adhesion energy and adsorption energy

The interface adhesion energy was computed with *E*_ad_=2*E*_GR/GO_+*E*_C*x*N*y*_−*E*_sandwich_, where *E*_GR/GO_, *E*_C*x*N*y*_ and *E*_sandwich_ represent the energies of the GR/GO, g-C_*x*_N_*y*_ and sandwich complex, respectively. The adsorption of water to GR_F_ in neutral system was calculated with *E*_ads_=*E*_GRF_+*E*_H2O_−*E*_H2O@GRF_ (*E*_GRF_, *E*_H2O_, *E*_H2O@GRF_ represent energies of the separated parts and their complex).

### AI-NAMD details

As for the AI-NAMD calculations^46^, we used density functional theory implemented by Vienna Ab initio Simulation Package package to carry out the *ab initio* molecular dynamics. The temperature was set to be 100 K and a 3.5 ps microcanonical trajectory is generated with a time step of 1 fs.

### Data availability

The authors declare that the data supporting the findings of this study are available within the article and its Supplementary Information files and from the corresponding author upon reasonable request.

## Additional information

**How to cite this article:** Yang, L. *et al*. Combining photocatalytic hydrogen generation and capsule storage in graphene based sandwich structures. *Nat. Commun.* **8,** 16049 doi: 10.1038/ncomms16049 (2017).

**Publisher’s note:** Springer Nature remains neutral with regard to jurisdictional claims in published maps and institutional affiliations.

## Supplementary Material

Supplementary Information

Supplementary Movie 1

Peer Review File

## Figures and Tables

**Figure 1:**
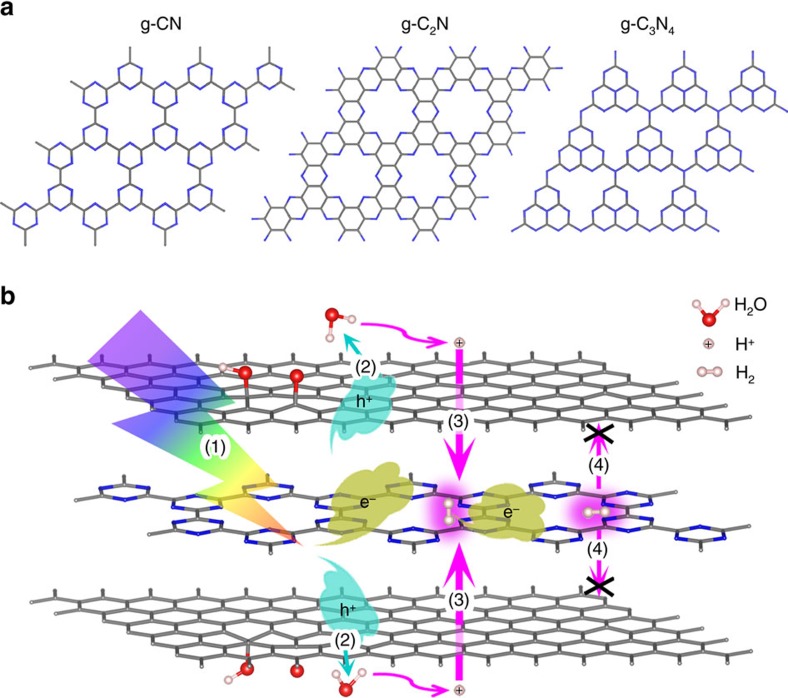
Scheme of water splitting and hydrogen capsuling scheme. (**a**) Models of graphitic carbon nitrides CN, C_2_N, C_3_N_4_. (**b**) The photocatalytic (water splitting) hydrogen generation and capsule storage scheme: (1) photo-generated electrons (e^−^) and holes (h^+^) separating; (2) water splitting to produce protons (H^+^) through holes (h^+^) attacking; (3) protons (H^+^) penetrating through GO and producing H_2_ molecules; (4) H_2_ molecules are prohibited from moving out of the sandwich. Here GO–CN–GO is used as an example. Blue, grey, pink and red beads stand for N, C, H (H^+^) and O atoms, the yellow and light blue clouds are for photo-generated electrons (e^−^) and holes (h^+^), and the blue and magenta arrows represent the migration of corresponding particles.

**Table 1:** Bader charge analysis of the sandwich structures.

**Neutral system GR/GO hole (h**^**+**^**)**	**GR**	**GO**_**OH**_	**GO**_**O**_
GR/GO–CN–GR/GO	0.34	0.72	0.54
GR/GO–C_2_N–GR/GO	0.19	0.20	0.18
GR/GO–C_3_N_4_–GR/GO	0.22	0.25	0.16
**1e^−^ induced C_*x*_N_*y*_ electron (e^−^)**	**CN**	**C_2_N**	**C_3_N_4_**
GR–C_*x*_N_*y*_–GR	−0.68	−0.45	−0.33
GO_OH_–C_*x*_N_*y*_–GO_OH_	−0.79	−0.33	−0.32
GO_O_–C_*x*_N_*y*_–GO_O_	−0.87	−0.41	−0.32
**1 h^+^ induced GR/GO hole (h^+^)**	**GR**	**GO_OH_**	**GO_O_**
GR/GO–CN–GR/GO	1.17	1.59	1.42
GR/GO–C_2_N–GR/GO	1.01	1.10	1.01
GR/GO–C_3_N_4_–GR/GO	1.20	1.23	1.11

The computed charge distributions on GR/GO and C_*x*_N_*y*_ layers in the neutral sandwich systems, and systems with one extra (photo-generated) electron and hole carriers. Here GO_OH_ and GO_O_ represent the hydroxyl and epoxy GO, respectively.
