# Supplementary material for: Combining photocatalytic hydrogen generation and capsule storage in graphene based sandwich structures
Source: Nat Commun. 2017 Jul 6;8:16049. doi: 10.1038/ncomms16049 (PMC5511497; doi:10.1038/ncomms16049)
Supplement: Supplementary Information [file ncomms16049-s1.pdf]

Title of file for HTML: Supplementary Information
Description: Supplementary Figures, Supplementary Tables and Supplementary References

Title of file for HTML: Supplementary Movie 1
Description: This movie file shows proton penetration process through the graphene sheet in the GR–C3N4 structure.

Title of file for HTML: Peer Review File
Description:

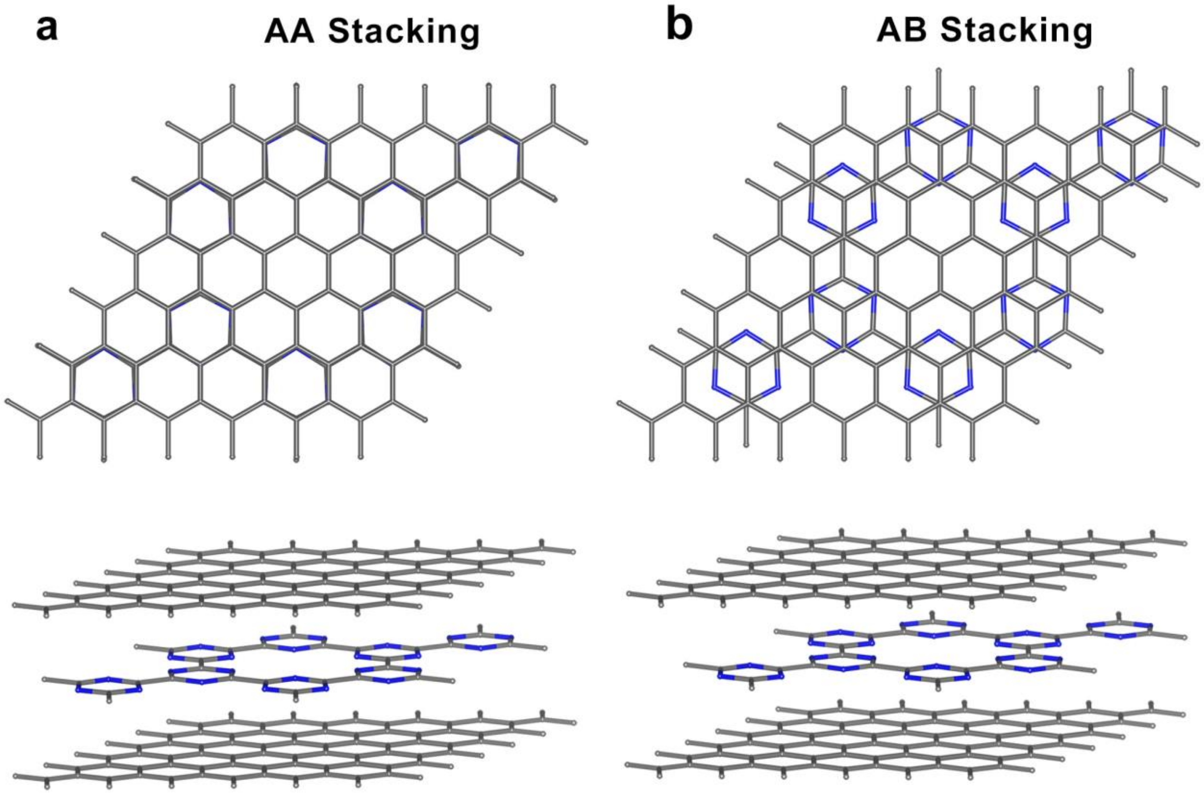

Supplementary Figure 1. Different stacking forms of the designed sandwich structures. Top and side view of the atomic structures of GR–CN–GR sandwich in the AA **(a)** and AB **(b)** stacking forms.

Here, AA stacking has all C and N atoms in g-C_xN_y layer right on top of one graphene carbon, while AB stacking means that half of the C or N atoms in g-C_xN_y locate over the centers of graphene hexagons. Our DFT calculations demonstrated that the AA stacking form for CN with graphene always hold larger total energy (ΔE= 0.38 eV) than the one with the AB form. It was also reported that the coupling of g-C_2N or g-C_3N_4 with graphene are more stable and stronger by taking the AB stacking form[1,2]. Therefore, we mainly focused on the AB stacking form in design of our sandwich systems.

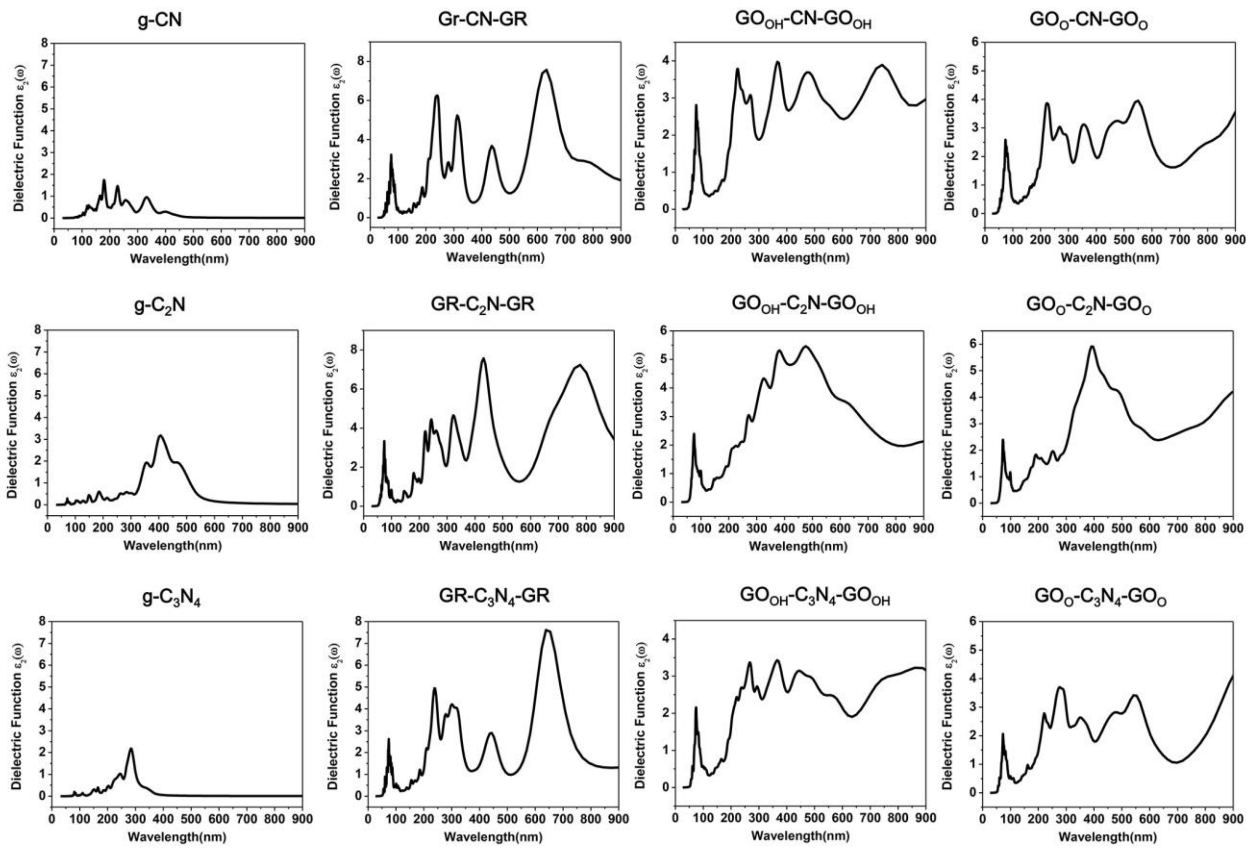

Supplementary Figure 2. Absorption spectrum of the C_xN_y and GR/GO–C_xN_y–GR/GO sandwich structures. The computed imaginary part of the dielectric function (reflecting photo-absorption ability) for the pure g-C_xN_y monolayer and the hybrid GR/GO–C_xN_y–GR/GO sandwich structures.

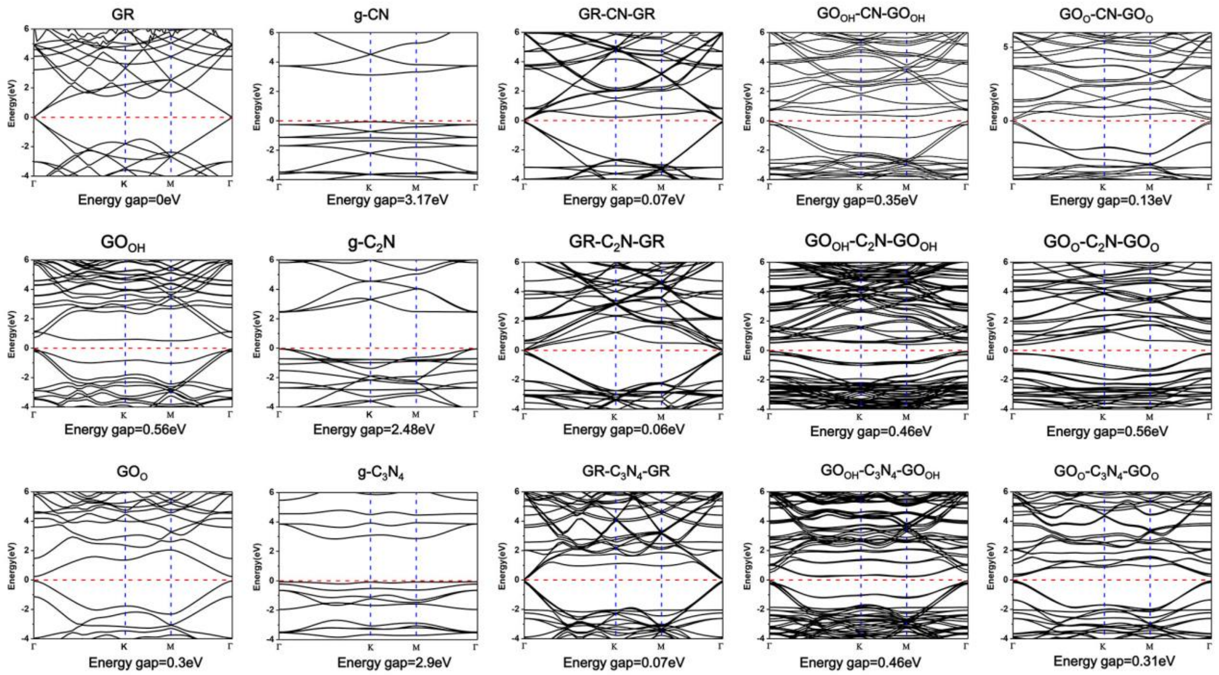

Supplementary Figure 3. The energy band structures of the bare GR/GO, C_xN_y, and GR/GO–C_xN_y–GR/GO materials. The computed energy band structures of the bare GR, GO, CN, C_2N, C_3N_4 and the sandwiched GR/GO–C_xN_y–GR/GO at the HSE06 functional level. The Fermi level is set at 0 eV, and the dotted blue lines represent the high symmetry positions for K, M points.

The energy band structures of the bare CN, C_2N, C_3N_4 are basically consistent with the previous report[3-5]. And the sandwiched GR–C_xN_y–GR structures all come up with the band gap opening in the graphene due to the interaction of the graphene and C_xN_y[1,2,4].

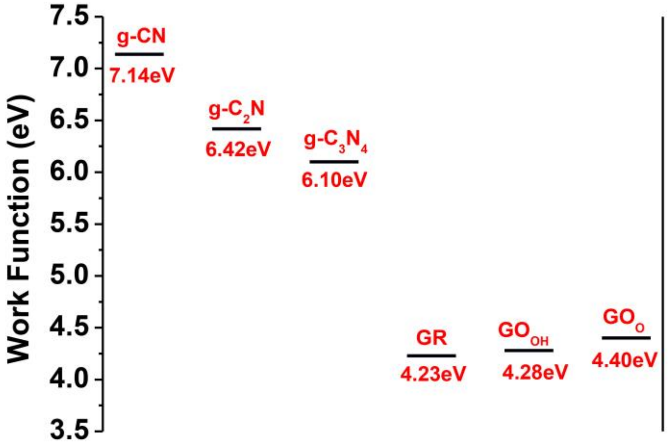

Supplementary Figure 4. The work functions of the bare GR/GO and C_xN_y structures. The computed work functions of the bare GR, GO_{OH}, GO_{O}, CN, C_2N, C_3N_4 at the HSE06 functional level.

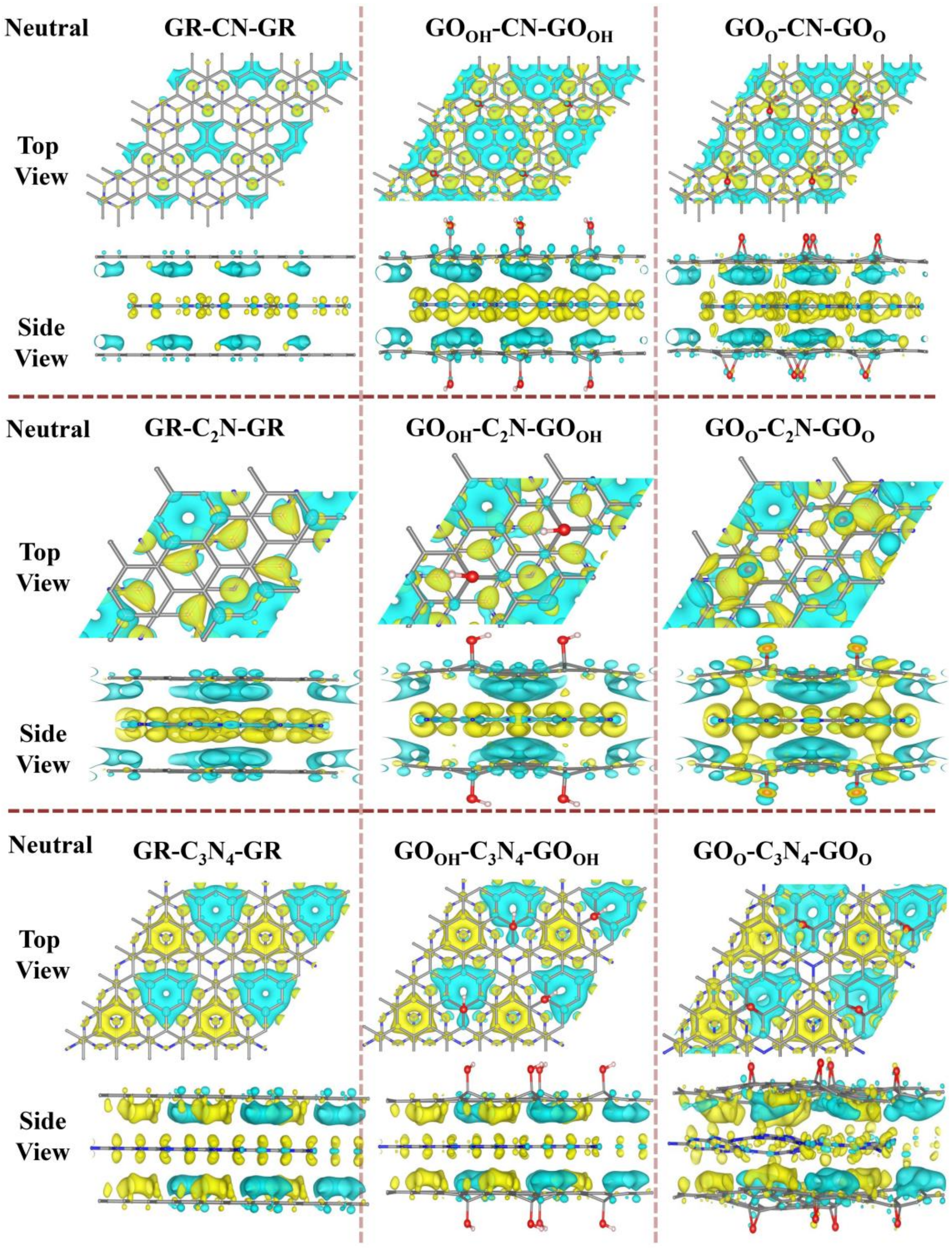

Supplementary Figure 5. Charge density difference of the neutral sandwich structures. Charge distribution computed as Bader charge differences between the neutral GR/GO–C_xN_y–GR/GO sandwich structures and the bare monolayers of GR/GO and g-C_xN_y, from top and side view. Yellow and blue bubbles represent electron and hole charges, and the isosurface values for

the GR/GO–CN–GR/GO, GR/GO–C_2N–GR/GO, GR/GO–C_3N_4–GR/GO are 0.0005 e/Å^3, 0.0007 e/Å^3 and 0.0004 e/Å^3, respectively.

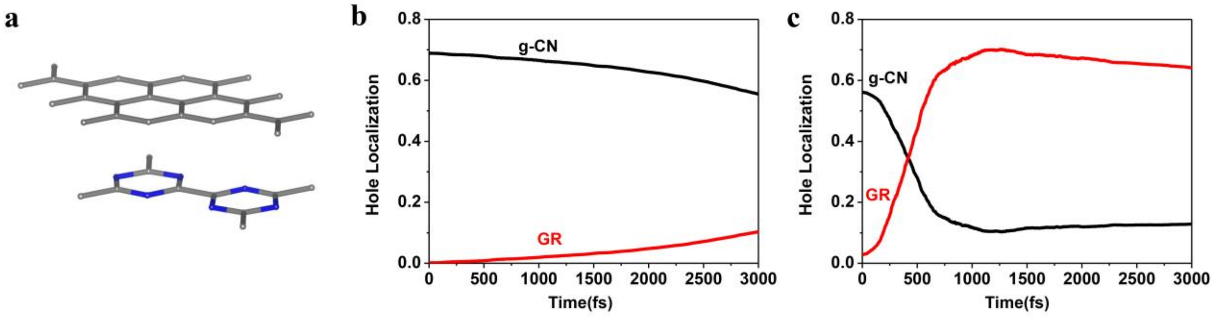

Supplementary Figure 6. Ultrafast hole evolution between the GR-CN layers. **(a)** Optimized configuration of the GR–CN hybrid structure for the *ab initio* non-adiabatic molecular dynamics (AI-NAMD) calculation. **(b)** Time dependent spatial hole localization at the Γ points in the GR–CN sheet at 100 K for holes with lower energy (near the valence band maximum). **(c)** Time dependent spatial hole localization at the Γ points in the GR–CN sheet at 100 K for holes with higher energy.

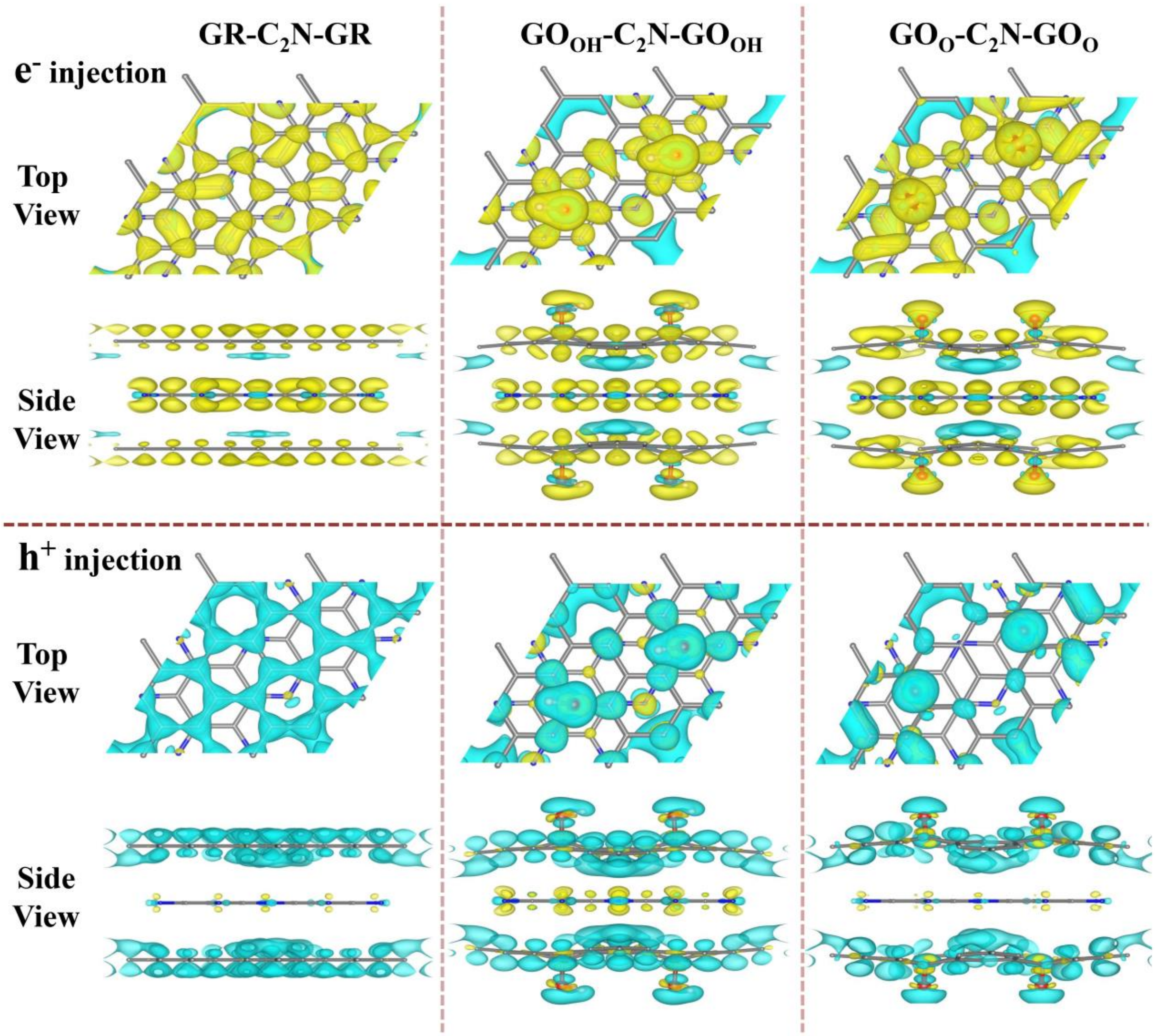

Supplementary Figure 7. Photo-generated carriers distributions for the GR/GO–C_2N–GR/GO sandwich structures. Charge distribution computed as Bader charge differences between GR/GO–C_2N–GR/GO sandwich with one extra carrier (photo-generated electron (e^-) or hole (h^+)) and the neutral monolayers of GR/GO and g-C_2N, from top and side view. Yellow and blue bubbles represent electron and hole charges with isosurface value of 0.0007e/$Å^3$.

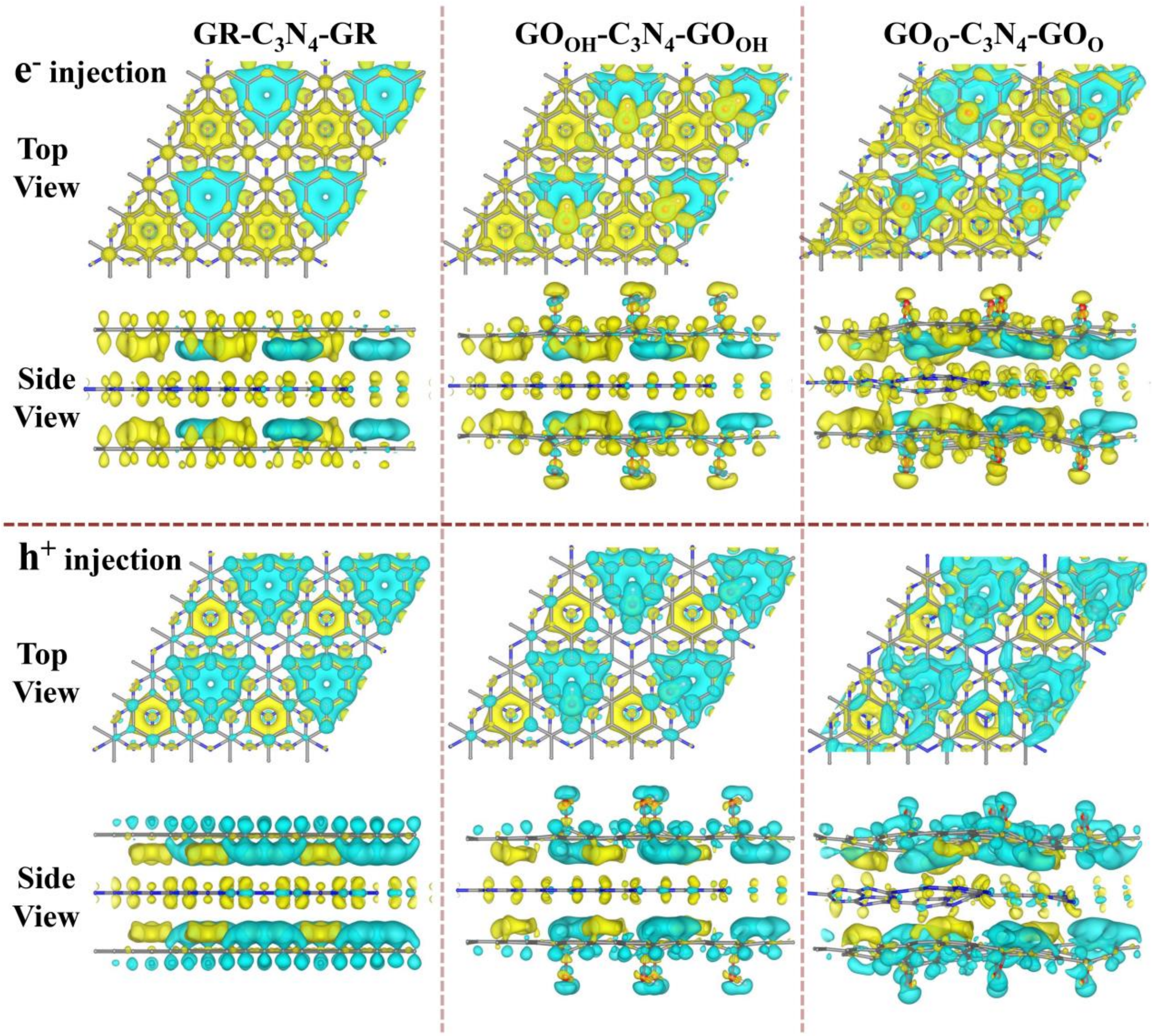

Supplementary Figure 8. Photo-generated carriers distributions for the GR/GO–C_3N_4–GR/GO sandwich structures. Charge distribution computed as Bader charge differences between GR/GO–C_3N_4–GR/GO sandwich with one extra carrier (photo-generated electron (e^-) or hole (h^+)) and the neutral monolayers of GR/GO and g-C_3N_4, from top and side view. Yellow and blue bubbles represent electron and hole charges with isosurface value of 0.0004 e/Å^3.

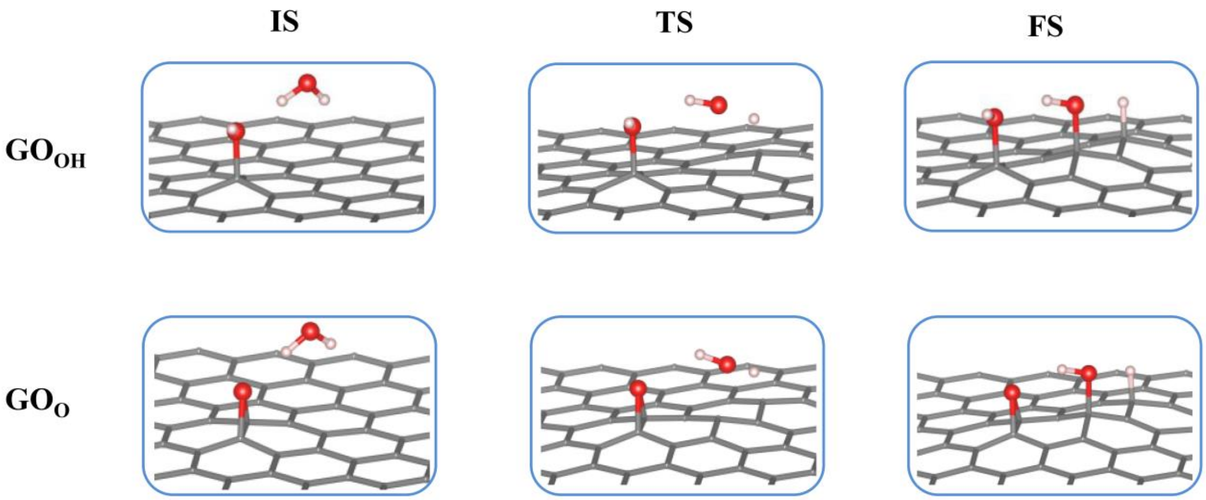

Supplementary Figure 9. NEB transition state calculations for water splitting at the outer GO surface. The reaction path for water molecule catalyzed by GO_{OH} and GO_{O} sheet. Here GO_{OH} and GO_{O} represent the hydroxyl and epoxy GO, respectively.

Note here the initial state configurations are slightly different to the most stable structure in Fig. 3a and 3b with energy differences $< \sim 0.08$ eV, which are more favorable for CI-NEB searching of transition states.

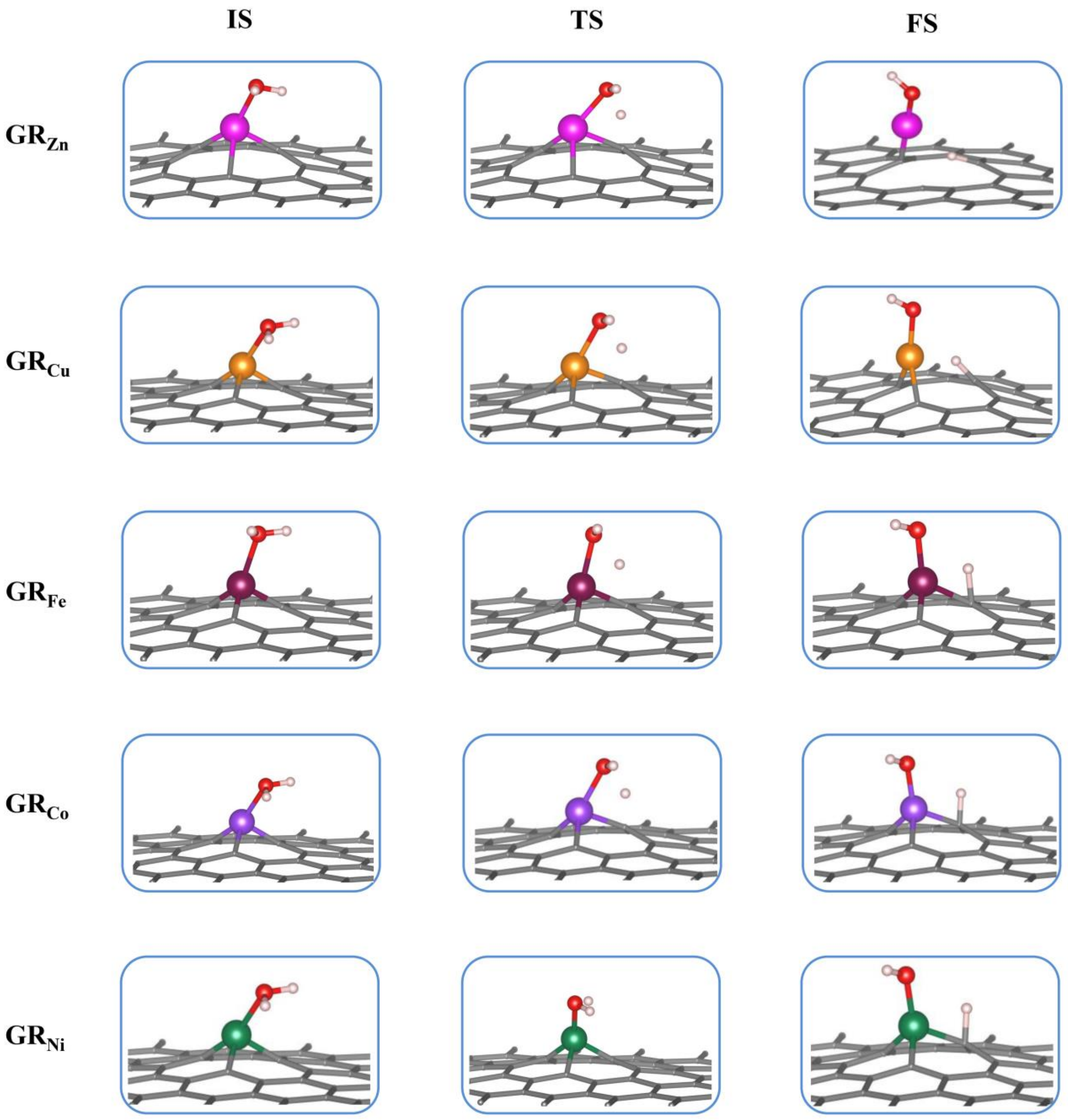

Supplementary Figure 10. NEB transition state calculations for water splitting at the outer metal doped GR surface. The reaction path for water molecule catalyzed by GR_{Zn}, GR_{Cu}, GR_{Fe}, GR_{Co}, GR_{Ni} sheets. Here the magenta, orange, mulberry, purple and green beads represent the metal atom of Zn, Cu, Fe, Co, Ni.

The initial configurations of the metal-doped GR sheets were based on reported literatures[6,7].

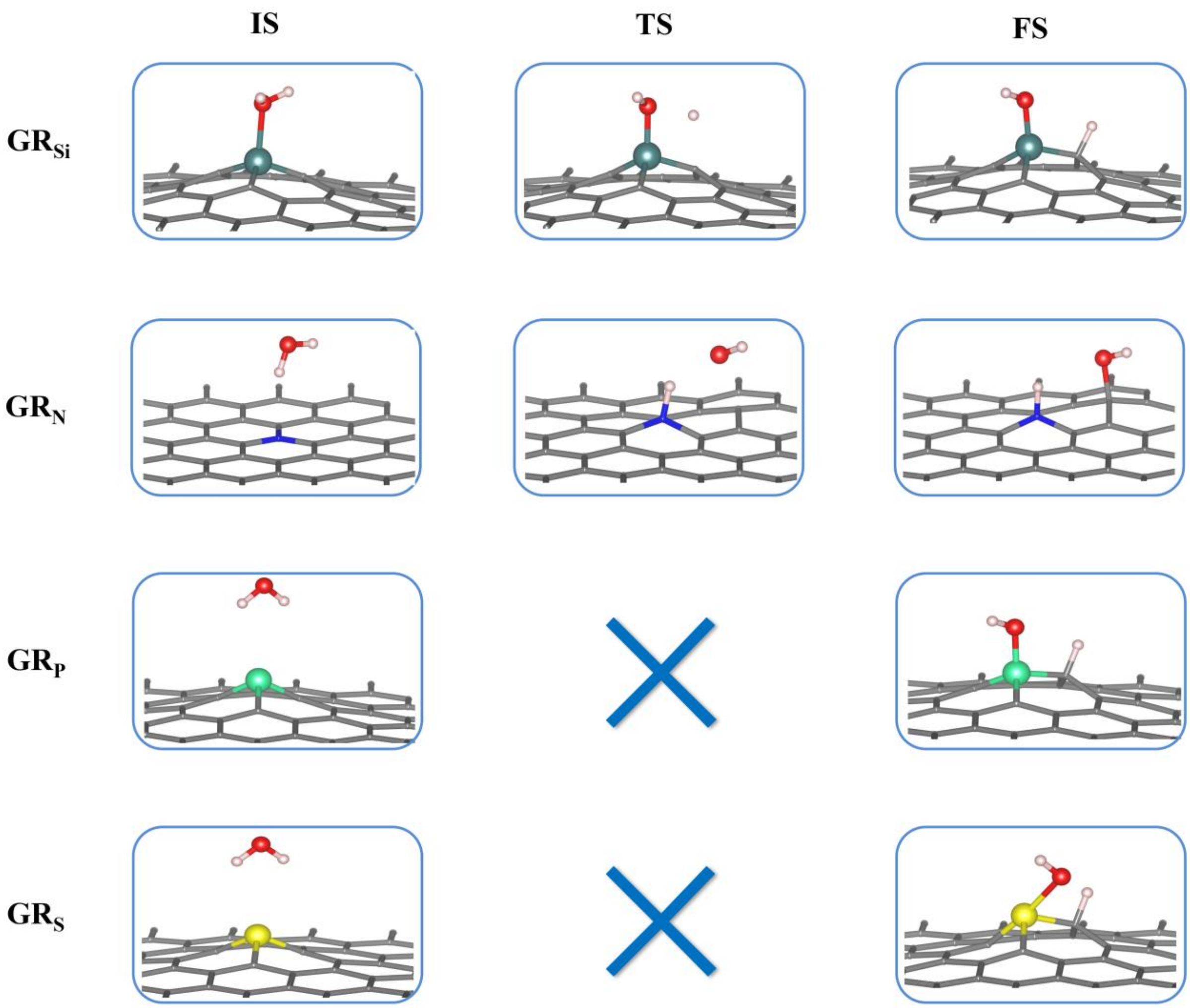

Supplementary Figure 11. NEB transition state calculations for water splitting at the non-metal doped GR surface. The reaction path for water molecule catalyzed by GR_{Si}, GR_N, GR_P, GR_S sheets. Here the dark cyan, blue, grass green and yellow beads represent the atoms of Si, N, P, S. The transition states were not found for GR_P and GR_S.

The initial configurations of the non-metal doped GR sheets were based on previous literatures[8,9].

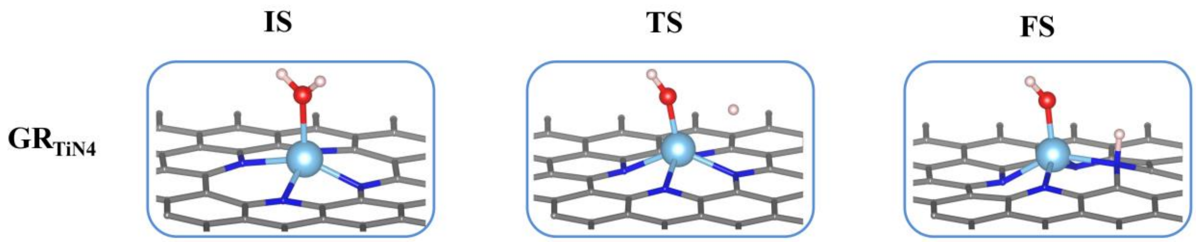

Supplementary Figure 12. NEB transition state calculations for water splitting at the TiN_4-embedded GR surface. The reaction path for water molecule catalyzed by GR_{TiN4} sheet. Here the sky-blue bead represents Ti atom.

The initial configuration of water molecule adsorbed to the GR_{TiN4} sheet was based on previous literatures[10].

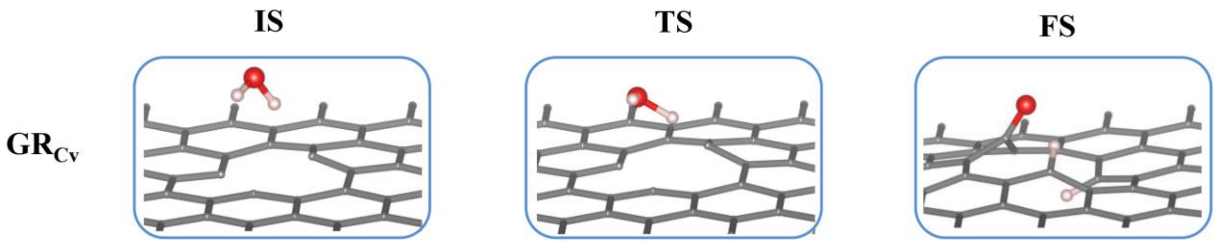

Supplementary Figure 13. NEB transition state calculations for water splitting at the defected GR surface. The reaction path for water molecule catalyzed by GR_{Cv} sheet. Here C_v is the abbreviation of the carbon vacancy.

The initial configuration of the water molecule adsorbed to GR_{Cv} sheet was based on previous literatures[11].

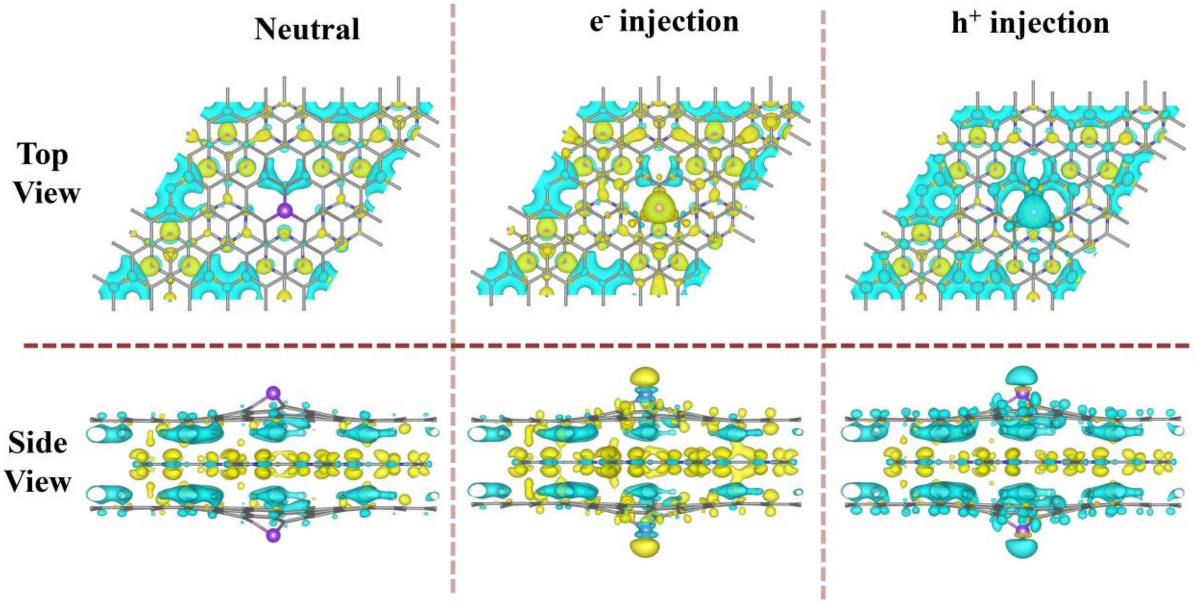

Supplementary Figure 14. Charge distributions of the GR_{Co}–CN–GR_{Co} sandwich structure. Charge distribution computed as Bader charge differences between the GR_{Co}–CN–GR_{Co} sandwich structures and the bare monolayers of GR_{Co} and g-CN, together with the charge differences between GR_{Co}–CN–GR_{Co} sandwich with one extra carrier (photo-generated electron (e-) or hole (h+)) and the neutral monolayers of GR_{Co} and g-CN, from top and side views. Here yellow and blue bubbles represent electron and hole charges with isosurface value of 0.0005 e/Å^3.

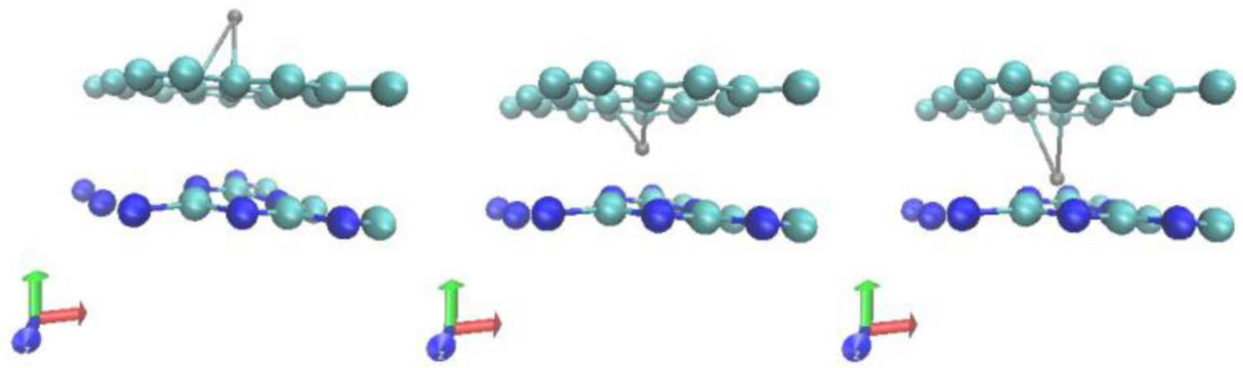

Supplementary Figure 15. Proton penetrates through the graphene sheet in the GR-C_3N_4 structure. Schematic figures of the ab initio MD simulations for the proton penetration through the graphene sheet to meet the C_3N_4 layer in the GR–C_3N_4 structure. Here the cyan, blue and grey beads represent carbon, nitrogen, hydrogen atoms, respectively. The full dynamic process of proton transfer is given in the supplementary file of Supplementary Movie 1.

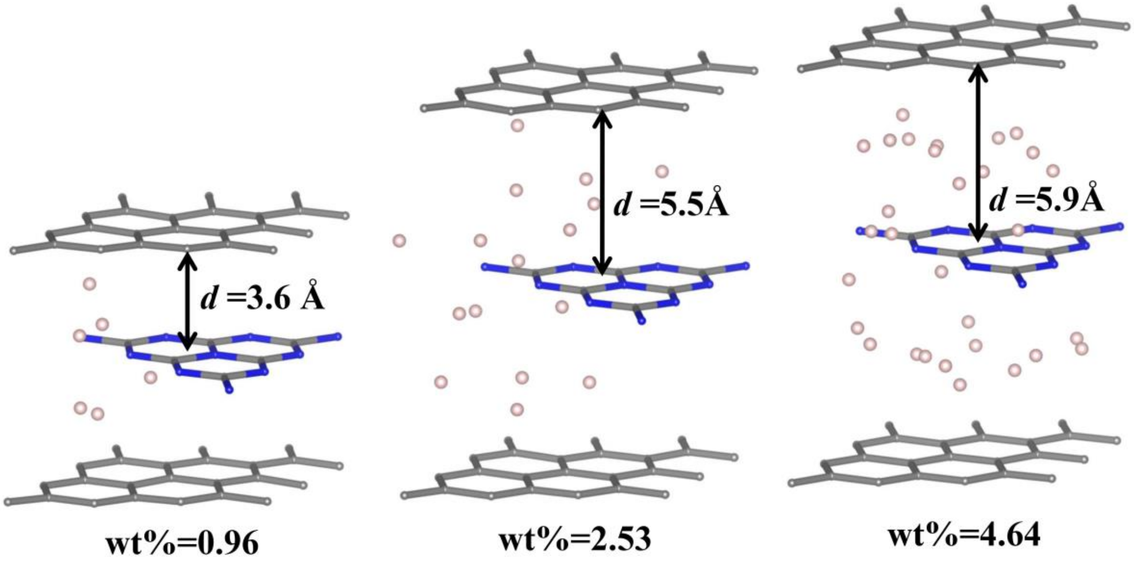

Supplementary Figure 16. Hydrogen storage of the GR−C_3N_4−GR sandwiched structure. Optimized configurations of the GR–C_3N_4–GR sandwich structure adsorbed with H_2 molecules at the storage rate of 0.96 wt%, 2.53 wt% and 4.64 wt% with different interfacial spaces.

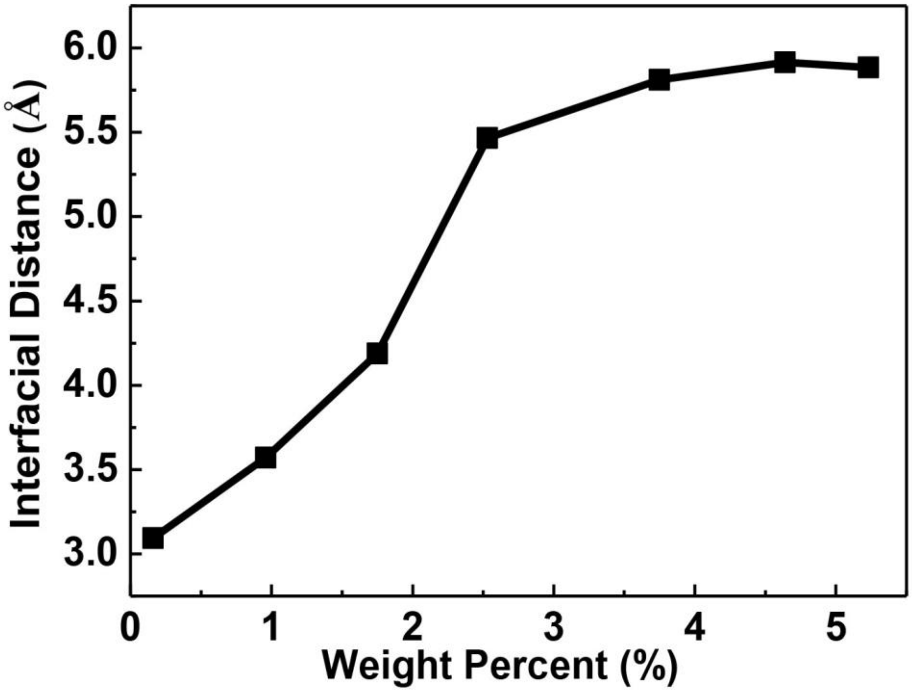

Supplementary Figure 17. Interfacial distance for different H_2 store rate. The variation of the equilibrium interfacial distance to achieve different H_2 store rate when full structural relaxation is allowed.

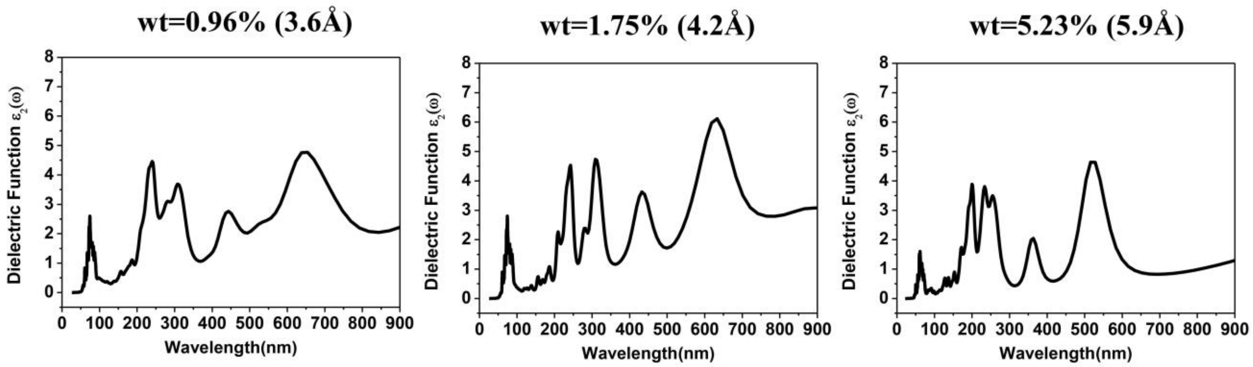

Supplementary Figure 18. Absorption spectrum of the GR−C_3N_4−GR sandwiched structures with the addition of hydrogen. The computed imaginary part of the dielectric function (reflecting photo-absorption ability) for the GR–C_3N_4–GR sandwiched structures with the hydrogen storage rate at 0.96 wt% (interlayer distance 3.6 Å), 1.75 wt% (interlayer distance 4.2 Å), and 5.23 wt% (interlayer distance 5.9 Å).

Simulations demonstrated that the sandwiched structure is also efficient in harvesting visible and ultraviolet light with the addition of hydrogen.

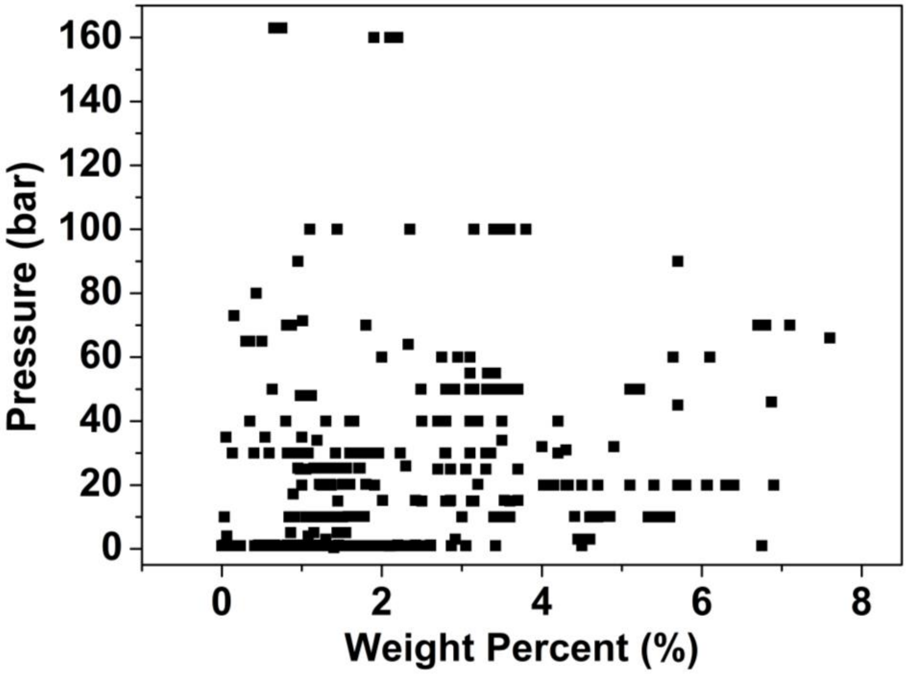

Supplementary Figure 19. H_2 storage rated achieved in literature. The pressurization applied by previously reported materials for effective hydrogen storage. 1 bar=10^5 pa (~ 1 standard atmospheric pressure). Data points are retrieved from http://www.hydrsm.csdb.cn/index.html.

Supplementary Table 1. Interface adhesion energy and equilibrium distance of the GR/GO–C_xN_y–GR/GO sandwich structures. The interface adhesion energy (E_{ad}) and equilibrium distance (d_{layer}) between the CN, C_2N, C_3N_4 and GR/GO layers in the sandwich structures. Here GO_{OH} and GO_O represent the hydroxyl and epoxy GO, respectively.

		GR/GO–CN–GR/GO	GR/GO–C_2N–GR/GO	GR/GO–C_3N_4–GR/GO
E_{ad} (eV)	GR	2.58	1.13	1.04
	GO_{OH}	2.60	1.11	3.37
	GO_O	2.80	1.02	3.73
d_{layer} (Å)	GR	3.40	3.20	3.09
	GO_{OH}	3.05	3.14	3.12
	GO_O	3.10	3.15	2.94

Supplementary Table 2. Adsorption energies of water on GR_F materials and corresponding energy barriers for water splitting. The computed water adsorption energies (E_{ads}) and water splitting energy barriers (E_b) for water on GR_F with functional groups of doped heteroatoms and defect.

Energy(eV)		E_{ads}	E_b
GO	GO_{OH}	0.37	3.34
	GO_O	0.32	3.56
Metal atom doped GR	GR_{Zn}	0.60	0.58
	GR_{Cu}	0.78	0.66
	GR_{Fe}	1.02	0.87
	GR_{Co}	0.82	1.09
	GR_{Ni}	0.77	1.52
Non-metal atom doped GR	GR_{Si}	0.47	0.41
	GR_N	0.07	3.57
TiN_4 doped GR	GR_{TiN4}	1.05	0.86
Defected GR	GR_{Cv}	0.20	0.40

Supplementary Table 3. Bader charge analysis of the sandwich system of GR_{Co}–CN–GR_{Co} structures. The computed charge distributions on GR_{Co} and g-CN layers in the neutral sandwich systems, and systems with one extra (photo-generated) electron and hole carriers.

GR_{Co} (in neutral)	CN (in 1e^- system)	GR_{Co} (in 1 h^+ system)
0.50 h^+	-0.66 e^-	1.41 h^+

The hybrid system made of GR_F and g-CN can achieve effective charge separation, similar to those of GR/GO–C_xN_y–GR/GO sandwich structures.

Supplementary Table 4. Gibbs free energy changes for the water splitting reaction. The computed Gibbs free energies for the water splitting reaction in the neutral and 1 h^+ injection systems.

neutral system	GO_{OH}	GO_O
ΔG (eV)	1.55	2.34
$1h^+$ injection	GO_{OH}	GO_O
ΔG (eV)	1.08	1.67

The reaction step for the water molecule absorbed to GO in our system is:

$$* + H_2O \rightarrow *OH + (H^+ + e^-)$$

* stands for the GO sheet, $\Delta G = \Delta E + \Delta ZPE - T\Delta S - \Delta G_{pH} + 1/2G_{H2} - eU$[12], $\Delta G_{pH} = 2.303k_BT \times pH$ (k_B, the Boltzmann constant, T, the temperature, and PH=0 in our system), U is the applied potential with respect to the normal hydrogen electrode (NHE), in our system, U=0 (without any external potential). ΔE represents the reaction energy by DFT calculations, ΔZPE is the zero point energy by harmonic vibrational frequency calculations, ΔS is the entropy difference between the adsorbed state and the gas phase. The value of TS for H_2O is 0.67 eV[13].

Supplementary Table 5. Coulomb interaction energy of proton due to the attraction of C_xN_y sheet in GR/GO–C_xN_y–GR/GO sandwich structures. The coulomb interaction energy of proton is produced by the attraction of C_xN_y sheet with photo-generated electrons in GR/GO–C_xN_y–GR/GO sandwich structures. Here GO_{OH} and GO_O represent the hydroxyl and epoxy GO, respectively.

Coulomb interaction energy (eV)	GR/GO–CN–GR/GO	GR/GO–C_2N–GR/GO	GR/GO–C_3N_4–GR/GO
GR	2.88	2.02	1.54
GO_{OH}	3.73	1.51	1.48
GO_O	4.04	1.87	1.56

Our ab initio MD simulations show the proton transfer process through the graphene sheet in the GR-C_3N_4 structure (Supplementary Movie 1, Supplementary Fig. 15), confirming the proton penetration in our system. To describe it quantitatively, we calculated the electrostatic interaction energy between the proton and the C_xN_y with photo-generated electrons in the sandwiched structures. The smallest coulomb interaction energy is 1.48 eV at the optimized interfacial distance, exceeding the proton penetration barrier through graphene of 1.23 eV. We need to point out that the origin vacuum between the GO and C_xN_y sheet is only 2.94~3.26 Å for all of the systems. The interfacial distance could be increased to ~5.0 Å after hydrogen storage, which results in coulomb interaction energy of 0.92~2.51 eV, and is still sufficient for overcoming the proton penetration barrier.

Supplementary Table 6. Bader charge analysis for the GR–C_3N_4–GR sandwich structures with the interfacial distance of 3.1 ~ 5.9 Å. The computed charge distributions on GR and C_3N_4 layers in the system of GR–C_3N_4–GR with one extra (photo-generated) electron and hole carriers at the interfacial distance of 3.1, 3.6, 4.2 and 5.9 Å.

1e$^-$ induced C_3N_4 Electron (e$^-$)	C_3N_4 (3.1 Å)	C_3N_4 (3.6 Å)	C_3N_4 (4.2 Å)	C_3N_4 (5.9 Å)
GR–C_3N_4–GR	-0.27	-0.26	-0.16	-0.16
**1h$^+$ induced GR Hole (h$^+$)**	**GR (3.1 Å)**	**GR (3.6 Å)**	**GR (4.2 Å)**	**GR (5.9 Å)**
GR–C_3N_4–GR	0.97	0.92	0.88	0.70

Originally, the interfacial distance is at 3.1 Å, when one photo-generated electron carrier could induce about 0.27 e$^-$ in the C_3N_4 sheet, while the GR cells can collect 0.97 h$^+$ with one extra hole injection. As for the interfacial distance of 3.6~5.9 Å, although the hole charges accumulated by graphene sheet decrease as the interfacial distance increases, there are still 70~92% of one extra injected hole being localized on the graphene surface. This is the indicative of effective electron-hole separation in the hybrid structure with the enlarged interfacial distance.

Supplementary References

1. Du, A. J. *et al*. Hybrid graphene and graphitic carbon nitride nanocomposite: gap opening, electron–hole puddle, interfacial charge transfer, and enhanced visible light response. *J. Am. Chem. Soc.* **134,** 4393–4397 (2012).
2. Wang, D. D., Han, D. X., Liu, L and Niu, L. Structure and electronic properties of C_2N/graphene predicted by first-principles calculations. *RSC Adv*. **6,** 28484–-28488 (2016).
3. Li, X. Y. *et al*. Graphitic carbon bitride supported single-atom catalysts for efficient oxygen evolution reaction. *Chem. Commun*. **52**, 13233–13236 (2016).
4. Srinivasu, K., Modak, B., Ghosh, S. K. Porous graphitic carbon nitride: a possible metal-free photocatalyst for water splitting. *J. Phys. Chem. C* **118,** 26479–26484 (2014).
5. Liu, J. J. Origin of high photocatalytic efficiency in monolayer g−C_3N_4/CdS heterostructure: a hybrid DFT study. *J. Phys. Chem. C* **119,** 28417−28423 (2015).
6. Deng, D. H. *et al*. Catalysis with two-dimensional materials and their heterostructures. *Nat. Nanotechnol.,* **11,** 218–230 (2016).
7. Qiu, H. J. *et al.* Nanoporous graphene with single-atom nickel dopants: an efficient and stable catalyst for electrochemical hydrogen production. *Angew. Chem. Int. Ed.* **127,** 14237–14241(2015).
8. Chen, Y. *et al*. Silicon-doped graphene: an effective and metal-free catalyst for NO reduction to N_2O? *ACS Appl. Mater. Interfaces* **5,** 5994−6000 (2013).
9. Wang, H. B., Maiyalagan, T., Wang, X. Review on recent progress in nitrogen-doped graphene: synthesis, characterization, and its potential applications. *ACS Catal.* **2,** 781–794 (2012).
10. Liu, L. L., Chen, C. P., Zhao, L. S., Wang. Y., Wang, X. C. Metal-embedded nitrogen-doped graphene for H_2O molecule dissociation. *Carbon* **115,** 773–780 (2017).
11. Xu, Z. *et al*. Reversible hydrophobic to hydrophilic transition in graphene via water splitting induced by UV irradiation. *Sci. Rep.* **4,** 6450 (2014).
12. Heyd, J., Scuseria G. E and Ernzerhof, M. Hybrid functionals based on a screened coulomb potential. *J. Chem. Phys.* **118,** 8207–8215 (2003).
13. Zhuo, Z. W., Wu, X. J., Yang, J. L. Two-dimensional phosphorous porous polymorphs with tunable band gaps. *J. Am. Chem. Soc.* **138,** 7091–7098 (2016).
