# Supplementary figures and images for: Combining photocatalytic hydrogen generation and capsule storage in graphene based sandwich structures
Source: Nat Commun. 2017 Jul 6;8:16049. doi: 10.1038/ncomms16049 (PMC5511497; doi:10.1038/ncomms16049)

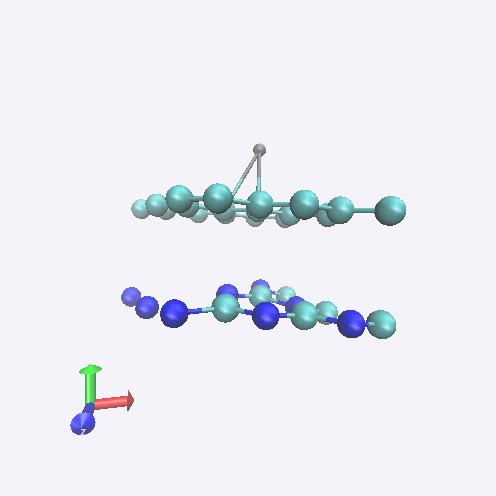

Supplement: Supplementary Movie 1 [file ncomms16049-s2.gif]
